# Novel maturity parameters for mature to over-mature source rocks and oils based on the distribution of phenanthrene series compounds

**DOI:** 10.1016/j.heliyon.2016.e00085

**Published:** 2016-03-08

**Authors:** Zixiang Wang, Yongli Wang, Baoxiang Wu, Gen Wang, Zepeng Sun, Liang Xu, Shenzhen Zhu, Lina Sun, Zhifu Wei

**Affiliations:** a Key Laboratory of Petroleum Resources, Gansu Province/Key Laboratory of Petroleum Resources Research, Institute of Geology and Geophysics, Chinese Academy of Sciences, Lanzhou 730000, PR China; b University of Chinese Academy of Sciences, Beijing, 100049, China

**Keywords:** Petrology, Petroleum geochemistry, Organic geochemistry

## Abstract

Pyrolysis experiments of a low-mature bitumen sample originated from Cambrian was conducted in gold capsules. Abundance and distribution of phenanthrene series compounds in pyrolysis products were measured by GC-MS to investigate their changes with thermal maturity. Several maturity parameters based on the distribution of phenanthrene series compounds have been discussed. The results indicate that the distribution changes of phenanthrene series compounds are complex, and cannot be explained by individual reaction process during thermal evolution. The dealkylation cannot explain the increase of phenanthrene within the EasyRo range of 0.9% ∼ 2.1%. Adding of phenanthrene into maturity parameters based on the methylphenanthrene isomerization is unreasonable, even though MPI 1 and MPI 2 could be used to some extent.

Two additional novel and an optimized maturation parameters based on the distribution of phenanthrene series compounds are proposed and their relationships to EasyRo% (x) are established: log(MPs/P) = 0.19x + 0.08 (0.9% < EasyRo% < 2.1%); log(MPs/P) = 0.64x − 0.86 (2.1% < EasyRo% < 3.4%); log(DMPs/TMPs) = 0.71x − 0.55 (0.9% < EasyRo% < 3.4%); log(MTR) = 0.84x − 0.75 (0.9% < EasyRo% < 3.4%). These significant positive correlations are strong argument for using log(MPs/P), log(DMPs/TMPs) and log(MTR) as maturity parameters, especially for mature to over-mature source rocks.

## 1 Introduction

Exploration for hydrocarbons in highly mature to over mature thermal stages is an important field in current global oil and gas exploration (Tissot and Welte, 1984; Liang and Chen, 2005; Walters et al., 2011; Wang and Han, 2011; Wei et al., 2010; Dutta et al., 2013). Recent breakthroughs have shown the significance of this research, including studies of the Sinian Dengying and lower Cambrian Longwangmiao reservoirs in the Sichuan Basin, South China (Huang and Wang, 2008; Zou et al., 2014a; Zou et al., 2014b) and the deep Lower Paleozoic reservoirs in the Tazhong Uplift, Tarim Basin, northwestern China (Li et al., 2015). However, the Sinian-Lower Paleozoic source rocks are generally of very high thermal maturity, and characterized by lacking of vitrinite (Dai et al., 2008; Dai et al., 2014; Zhang et al., 2007; Zhang et al., 2008; Leng et al., 2013; Wu et al., 2014). As a consequence, the vitrinite reflectance and most biomarker maturity parameters are infeasible to assess the maturity of Sinian-Lower Paleozoic source rocks and oils (Zhang et al., 2007; Zhang et al., 2008; Leng et al., 2013; Wu et al., 2014).

Aromatic hydrocarbons are important fraction in crude oil and source rock bitumen. The pathways of organic reactions could lead to the change in abundance and distribution of aromatic hydrocarbons in oils and ancient sediment extracts (Strachan et al., 1988; Radke et al., 1982a; Radke et al., 1982b; Alexander et al., 1985; Alexander et al., 1994). Due to the higher thermal stability of aromatic hydrocarbons, many aromatic hydrocarbons maturity parameters, which were based on the isomerization or alkylation–dealkylation processes of substituted phenanthrenes containing alkyl groups, had been hired to assess the maturity of source rock bitumens or crude oils with higher maturity (Stojanović et al., 2001; Stojanović et al., 2007; Wilhelms et al., 1998). However, some aromatic hydrocarbons maturity parameters only can be applied in a certain maturity range. For instance, the classical aromatic hydrocarbon maturity parameters - methyl phenanthrene index MPI 1 and MPI 2 show a reversal at higher thermal maturity (Radke et al., 1982a).

Naphthalene and phenanthrene series compounds, which are the most common component in aromatic hydrocarbons, are the major objects for aromatic hydrocarbons maturation parameters research (Stojanović et al., 2007). Phenanthrene series compounds (Ps) have greater thermal stability than naphthalene series compounds. Examination of a great number of crude oils and source rock bitumen, as well as the corresponding geosynthetic reactions, has shown that the abundance and distribution of Ps were controlled by isomerization and alkylation–dealkylation processes, generation and cracking processes of Ps (Chen et al., 2010). However, the abundance and local distribution of individual phenanthrene series compounds are susceptible to the origin of the organic matter (Fan et al., 1990), the presence of mineral catalysts in the source or reservoir rocks (Jovančićević et al., 1992; Jovančićević et al., 1993) and the mineral composition of the carrier system rocks. The whole distribution characteristics of Ps could be more effective to reflect the maturity. Therefore, it is necessary to study the relationship between the distribution characteristics of Ps and the thermal maturation.

In this paper, the distribution of Ps and their evolution with maturity have been investigated using a series of pyrolysis experiments for low-maturity bitumen originated from the Cambrian source rock, NW Sichuan Basin. The study aims to provide experimental evidence that will facilitate the development of novel aromatic hydrocarbons maturation indices, as well as to clarify limitations in the use of currently available indices, for application in studies of petroleum maturation.

## 2 Experimental

### 2.1 Sample

**Fig. 1:**
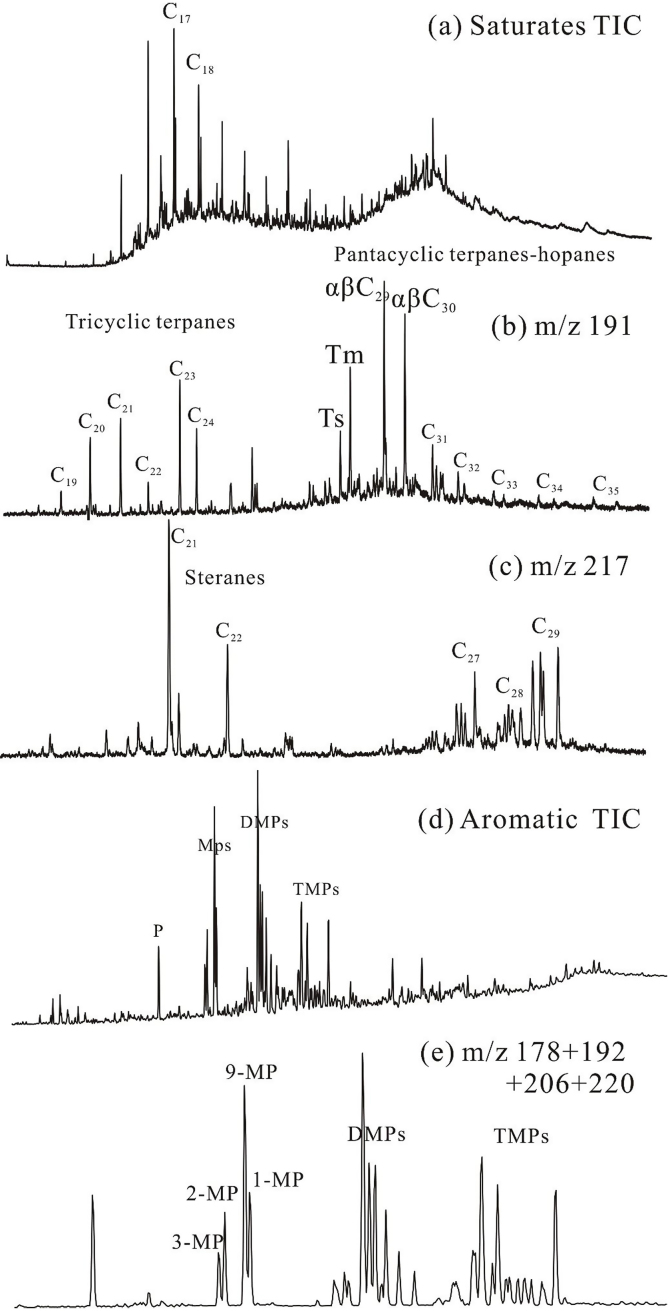
GC-MS total ion chromatogram (TIC) of the saturated fraction in EOM of bitumen (a; *n*-heptadecane and *n*-octadecane are indicated); m/z 191 mass chromatogram, displaying terpane distribution (b); m/z 217 mass chromatogram, displaying sterane distribution (c); GC-MS total ion chromatogram (TIC) of the aromatic fraction (d; Ns − naphthalenes; Ps − phenanthrenes; MPs − methylphenathrenes, DMPs − dimethylphenanthrenes; TMPs − trimethylphenanthrenes); (e) m/z 178 + 192 + 206 + 220 mass chromatograms of aromatic fraction, displaying phenanthrene series compounds distribution.

A sample of bituminous dike that originated from the Lower Cambrian black shale was collected from an outcrop in Kuangshanliang, northwest Sichuan Basin. The outcrop is located in a destroyed paleo-reservoir, and its organic geochemical properties and sources of bitumen have been reported (Zhang et al., 2014; Zhou et al., 2013). The abundance of extracted organic matter (EOM) is 8.3%, and the abundances of saturates, aromatics, resins and asphaltenes are 0.04%, 1.15%, 14.90% and 83.90%, respectively. The bitumen dike is depleted in low molecular weight hydrocarbons due to evaporation and biodegradation during exposure to the atmosphere (Huang and Wang, 2008). The maturity parameters Ts/Tm, MPI-1 and MPI-2 are 0.52, 0.47 and 0.57, respectively, suggesting that the bitumen sample has not undergone serious thermal degradation (Zhang et al., 2014; Zhou et al., 2013)(Fig. 1). The geochemical analysis of this bitumen shows that it is organic-rich, with a total organic carbon (TOC) of 58.0%, S1 of 2.42 mg HC/g sample, S2 of 36.96 mg HC/g sample, S3 of 0.40 mg CO_2_/g sample, HI of 637.06 mg/g TOC, Tmax of 401 °C and a δ^13^C value of −35.3‰.

A block of bituminous dike sample was pulverized and extracted for 72 h with chloroform using a Soxhlet apparatus. The yield of residual insoluble matter was 91.7%. We did not remove the inorganic matter, concerning that the TOC in sample is high and therefore the inorganic matter has little influence on hydrocarbon generation from kerogen. The obtained residual insoluble matter was used in pyrolysis experiments.

### 2.2 Pyrolysis experiments

The pyrolysis experiment was conducted in a confined system, following the procedures described in detail by Behar et al. (1992). Briefly, 15–60 mg of powder bitumen was loaded into a series of gold tubes (length, 40 mm; i.d., 5 mm; wall thickness, 0.5 mm). Then, the gold tubes were purged with argon for 5 min and sealed. The sealed gold tubes were placed in a series of autoclaves. The vessels were heated to 250 °C within 10 h and then from 250 °C to 600 °C at a rate of 2 °C/h. The pressure was maintained at 50 MPa. After heating, the autoclaves were removed from the oven and cooled in air. The gold tubes were cut off at room temperature (about 20 °C), and the samples were removed, wrapped with filter paper and extracted (36 h) in a Soxhlet apparatus with dichloromethane. Finally, the solvent was evaporated from the extracts, and the extracts were weighed.

### 2.3 Gas chromatography–mass spectrometry (GC–MS) analysis

The extracts were deasphalted by precipitation with n-hexane followed by filtration. The deasphalted maltenes were fractionated by column chromatography on alumina over silica gel. Saturated hydrocarbons, aromatic hydrocarbons and non-hydrocarbons were obtained by successively eluting with n-hexane, toluene and chloroform:methanol (98:2), respectively. The aromatic hydrocarbon fraction was analyzed using a 6890 N GC–5973 N mass spectrometer (GC–MS) equipped with a HP-5 capillary column (30 m × 0.32 mm i.d., 0.25 μm film thickness). The GC oven temperature was programmed from 80–300 °C at 4 °C/min and then held at this temperature for 30 min. Helium was used as carrier gas. MS conditions were EI ionization at 70 eV with an ion source temperature at 250 °C. The GC–MS system was operated in the full scan mode and scanned from m/z 50 to m/z 550.

### 2.4 Vitrinite reflectance

Sweeney and Burnham (1990) proposed a vitrinite maturation model (EasyRo%) to calculate the vitrinite reflectance Ro%. The model can be applied for temperature and time scales ranging from those in the laboratory to geological. In this study, the EasyRo% was hired as a calibration of thermal maturation for extrapolating the results of the high heating rate pyrolysis experiments to extremely low heating rate geological conditions. The calculated EasyRo% corresponding to heating temperature is shown in Table 1.

## 3 Results and discussion

### 3.1 Identification of phenanthrenes

**Fig. 2:**
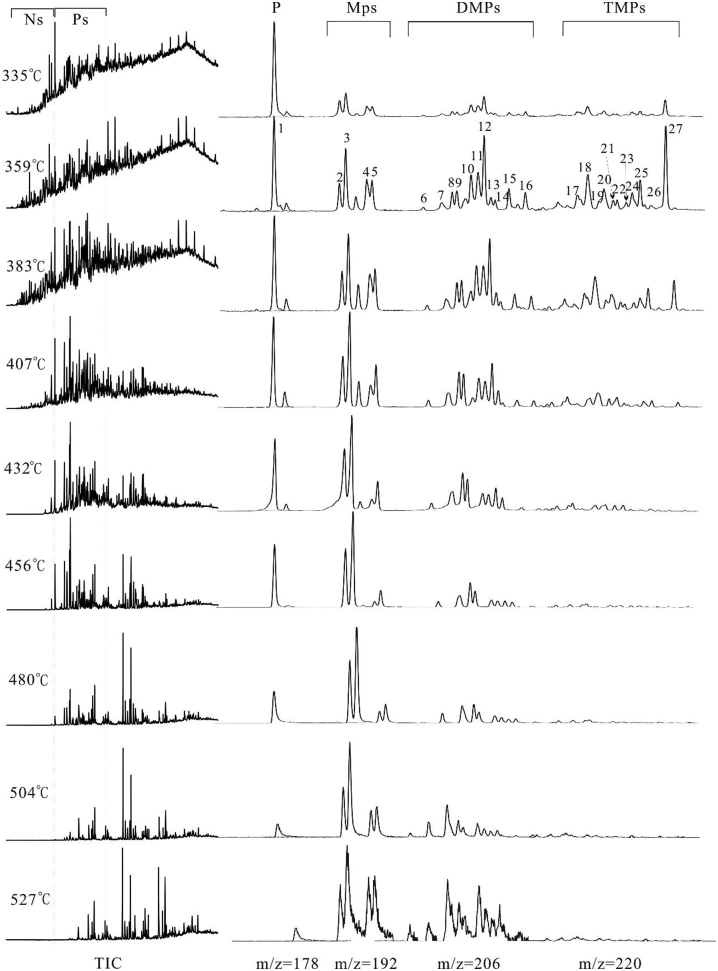
Total ion current (TIC) of aromatic hydrocarbons and mass chromatograms of P, MPs, DMPs, TMPs (m/z = 178 + 192 + 206 + 220) at different temperatures.

The Ps including phenanthrene (P), methylphenanthrenes (MPs), dimethylphenanthrenes (DMPs), trimethylphenanthrenes (TMPs) and tetramethylphenanthrenes (TeMPs) are the essential components nearly accounting for 4.08%–41.45% of the total aromatic hydrocarbons (Table 1). Each alkylphenanthrene series compounds (i.e. MPs, DMPs and TMPs) just include only the individual compounds shown in Fig. 2, and were identified in Table 2. The TeMPs have extremely low content, so that we could entirely ignore them in this paper.

Mass chromatograms of saturated and aromatic hydrocarbons are shown in Fig. 2. The relative abundances of phenanthrene series compounds are listed in Table 1.

### 3.2 Distribution of phenanthrene series compounds

**Fig. 3:**
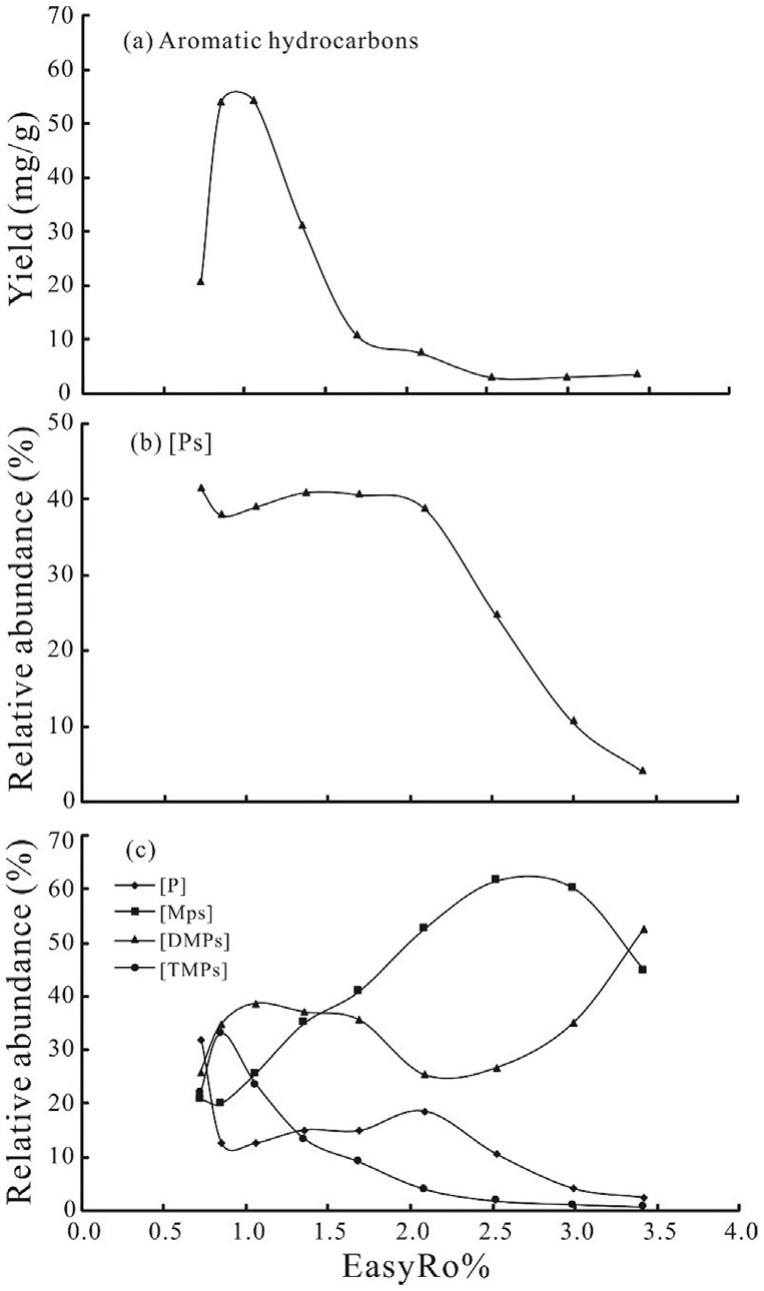
Changes in the yields of aromatic hydrocarbons (a) and relative abundances of Ps (b), P, MPs, DMPs and TMPs (c) with EasyRo%.

For each temperature point of the pyrolysis experiment, the corresponding EasyRo%, yields and relative abundance of different series compounds were obtained and discussed below. The relationships between calculated EasyRo%, and yields of aromatic hydrocarbons and the relative abundance of Ps in the aromatic hydrocarbons ([Ps]), as well as P, MPs, DMPs and TMPs in the Ps ([P], [MPs], [DMPs] and [TMPs]) are displayed in Table 1 and Fig. 3.

As shown in Fig. 3a, the yields of aromatic hydrocarbons progressively increase within the EasyRo range of 0.7% ∼ 1.0%, and then quickly decrease within the EasyRo range of 1.0% ∼ 3.4%. Given that Ps are the main compounds in aromatic hydrocarbons (Fig. 2), Ps may be mainly generated within the EasyRo range of 0.7% ∼1.0% and are destroyed within the EasyRo range of 1.0% ∼ 3.4%.

The [Ps] have little change within the EasyRo range 0.7% ∼ 2.1% (Fig. 3b), despite great changes that have taken place in the yields of aromatic hydrocarbons within the same EasyRo range (Fig. 3a). This suggests that the Ps have good thermostability in the range of EasyRo 0.7% ∼ 2.1%. Nevertheless, [Ps] quickly decrease within the EasyRo range 2.1% ∼ 3.4% indicating cracking of Ps.

Some aromatic maturation parameters, based only on the alkylation–dealkylation processes were successfully used for maturity assessment of crude oils and source rock bitumens (Stojanović et al., 2001; Stojanović et al., 2007). However, the influence of generation and decomposition of Ps were ignored when defining the aromatic hydrocarbons maturation parameters. [P] and [MPs] increase with maturity within the EasyRo% range 0.9% ∼ 2.1% and 0.9% ∼ 2.5%, respectively, and then decrease within the EasyRo% range 2.1% ∼ 3.4% and 2.5% ∼ 3.4%, respectively (Fig. 3c). However, the change of [DMPs] is quite different from [P] and [MPs]. [DMPs] firstly increase below the EasyRo% range of 1.1%, and then decrease within the EasyRo% range 1.1% ∼ 2.1%, and finally increase above the EasyRo% range of 2.1% (Fig. 3c). [TMPs] rapidly decrease with maturity above the EasyRo% of 0.9% (Fig. 3c). Apparently, these changes are complex, and cannot be explained by individual reaction process during thermal evolution.

In summary, in addition to the differences among the thermal stabilities of isomers and alkylation–dealkylation, the relative generation and decomposition rates of different Ps also influence their distribution during thermal maturation.

### 3.3 Evaluation of phenanthrene maturation parameters

In several earlier papers, crude oils and sedimentary bitumens have already been classified according to maturity parameters based on alkylphenanthrene alkylation–dealkylation reactions. Namely, some organic geochemical investigations (Stojanović et al., 2001; Stojanović et al., 2007; Simons et al., 2003; Singh et al., 1994), as well as geosynthetic modelling studies (Smith et al., 1995), have shown that during catagenetic evolution, depending on temperature and proton-donor type clay minerals, alkylation of phenanthrenes into methylphenanthrenes may have occurred, and also their dealkylation, particularly at higher degrees of maturity.

**Fig. 4:**
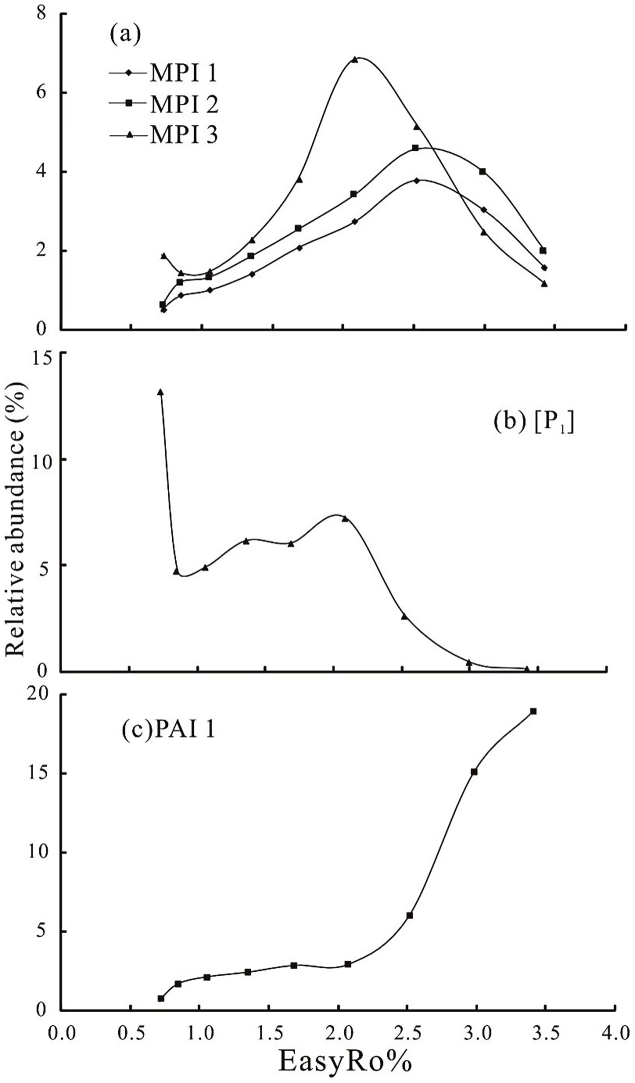
The variations of phenanthrene maturation parameters MPI 1, MPI 2, MPI 3 (a), [P1] (b) and PAI 1 (c) with EasyRo%.

**Fig. 5:**
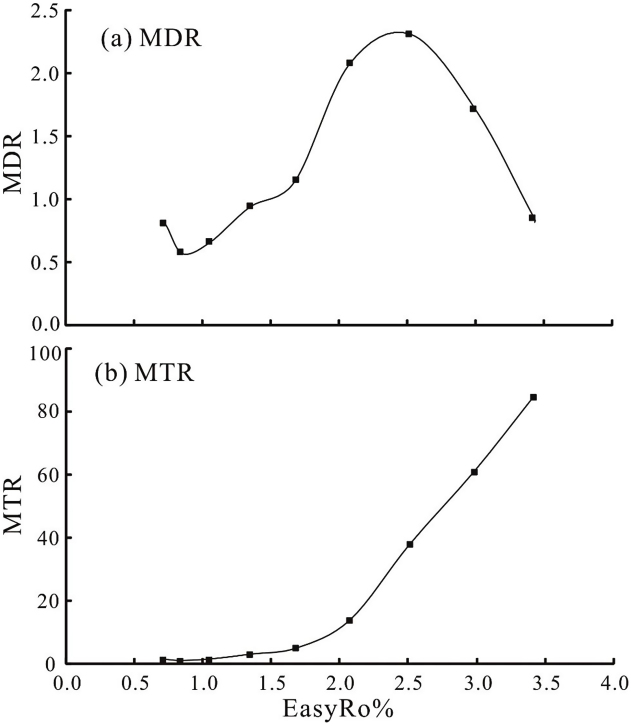
The variations of phenanthrene maturation parameters MDR (a) and MTR (b) with EasyRo%.

3-MP and 2-MP, which have a methyl group attached to a bridgehead position are more stable than 9-MP and 1-MP. Hence, the values of MPI-1 (methyl phenanthrene index, 1.5 × (2-MP + 3-MP)/(P + 9-MP + 1-MP)), MPI-2 (methyl phenanthrene index, 3 × 2-MP/(P + 9-MP + 1-MP)) and MPI-3 ((3-MP + 2-MP)/(9-MP + 1-MP)) were introduced to evaluate the maturity of crude oils and source rocks (Strachan et al., 1988; Radke et al., 1982a; Radke et al., 1982b; Alexander et al., 1985; Alexander et al., 1994). Some other parameters, such as [P1] (the concentration of phenanthrene in the tricyclic aromatic fraction) (Stojanović et al., 2001), PAI-1 ((3-MP + 2-MP + 9-MP + 1-MP)/P) (Ishiwatari and Fukushima, 1979), MDR (∑MP/∑DMP) and MTR (∑MP/∑TMP) (Stojanović et al., 2007), which were based on dealkylation reactions of alkyl-phenanthenes, were successfully used for maturity assessment of crude oils and source rock. In this paper, these parameters based on the relative concentrations of P, MPs, DMPs and TMPs are used to characterize the variations in abundance and distribution of Ps with increasing EasyRo% Table 3. Fig. 4 and Fig. 5 show the variation of phenanthrenes maturation parameters with EasyRo%.

MPI 1 and MPI 2 increase within the EasyRo range of 0.7% ∼ 2.5% and a reversal occurs above the EasyRo of 2.5% (Fig. 4a). Similarly, MPI 3, which is not affected by [P], progressively increases in the EasyRo range 0.9% ∼ 2.1%, and then quickly decreases within the EasyRo range of 2.1% ∼ 3.4% (Fig. 4a). This suggest that the isomerization of MPs mainly occurs within the EasyRo range of 0.9% ∼ 2.1%, and that the dominant factor affecting the distribution of methylphenanthrenes isomer have changed above EasyRo of 2.1%. The calculated formula of MPI 1 and MPI 2 include [P] in the denominator suggesting that [P] will decrease with the increase of thermal maturity. However, the pyrolysis experiment result show that [P] do not decrease within the EasyRo range of 0.9% ∼ 2.1% corresponding to isomerization stage of MPs (Fig. 4b). Hence, adding of phenanthrene into the methylphenanthrene isomerization maturity indicators within the EasyRo range of 0.9% ∼ 2.1% is unreasonable, even though MPI 1 and MPI 2 could be used to some extent (Bao et al., 1992).

Stojanović et al. (2001) suggested that together with methylphenanthrene isomerization in reservoir rocks, dealkylation also occurs, and the percentage of phenanthrene in the tricyclic aromatic fraction ([P1]) increase with maturity. Fig. 4b shows that [P1] increase within the EasyRo range of 0.9% ∼ 2.1%, while PAI 1 (MPs/P) slowly increase at the same EasyRo range (Fig. 4c). Apparently, the dealkylation cannot explain the increase of phenanthrene within the EasyRo range of 0.9% ∼ 2.1%.

As shown in Fig. 4c, PAI 1 slowly increases within the EasyRo range of 0.7–2.1%, and then rapidly increases above EasyRo of 2.1%. This contradicts the view that PAI 1 will decrease with the increase of thermal maturity (Ishiwatari and Fukushima, 1979; Stojanović et al., 2007). This conflicting result suggest that the change of PAI 1 for natural geological sample is different from pyrolysis experiment result, and may be affected by some other factors. Therefore, we need to be more careful, when we use the PAI 1 as a maturity parameter.

Stojanović et al. (2007) suggest that alkyl-phenanthrenes ratio MDR and MTR, which are based on demethylation of DMPs and TMPs into corresponding MPs, could be applied to assess the maturity of oils. Fig. 5 show that MDR increases from about 0.5 to 2.3 within the EasyRo range of 0.9% ∼ 2.5% and a reversal occurs above the EasyRo of 2.5%. MTR increases exponentially from the minimum at EasyRo of 0.9% to maximum 84.3 at EasyRo of 3.4%. This suggest that the MDR and MTR are useful in the EasyRo range of 0.9% ∼ 2.5% and 0.9% ∼ 3.4%, respectively, and the MTR is more sensitive to thermal maturation.

### 3.4 Novel maturation parameters based on the distribution of Ps

In fact, isomerization and alkylation–dealkylation, generation and decomposition of aromatic hydrocarbons may occur simultaneously and influence each other, and the effects degree of individual reaction process on the distribution of Ps is variable at different thermal stress. As a consequence, it is difficult to distinguish the dominant factor that affects the distribution of Ps. However, it is feasible to treat each alkyl series compounds as a whole and to investigate the influences of maturity process on the distribution of Ps, and to define new maturity parameters based on the evolution of distribution characteristics of Ps.

**Fig. 6:**
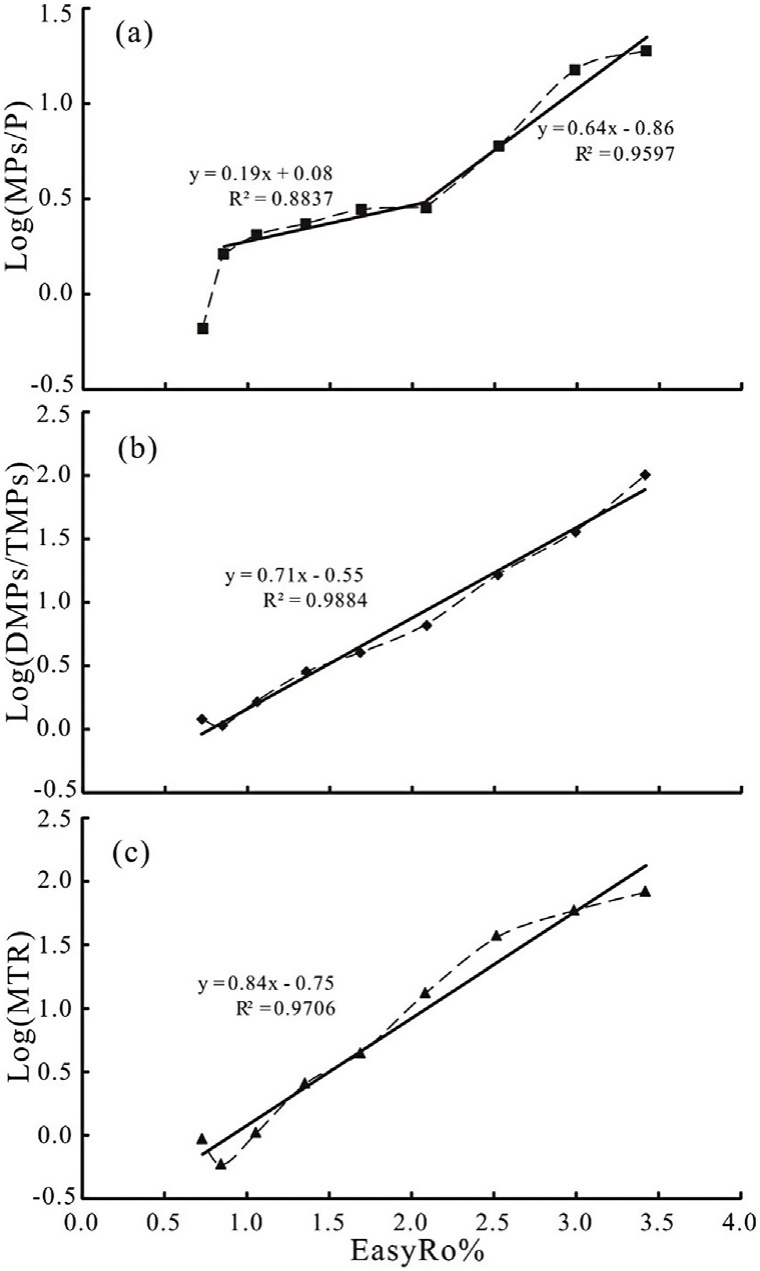
Relationships between EasyRo% and novel maturity parameters log(MPs/P) (a), log(DMPs/TMPs) (b) and log(MTR) (c).

Therefore, an optimized, two novel maturation parameters based on the distribution of Ps are proposed in this paper: log(DMPs/TMPs) and log(MPs/P), whereas MTR parameter is used in logarithmic form, log(MTR). Fig. 6 shows the relationship between novel maturation parameters and EasyRo%. All three maturation parameters have significant positive correlation with EasyRo%, and four equations with high correlation coefficients (R^2^) have been obtained:

log(MPs/P) = 0.19x + 0.08 (x = EasyRo%) (0.9% < EasyRo% < 2.1%, R^2^ = 0.8837)

log(MPs/P) = 0.64x − 0.86 (2.1% < EasyRo% < 3.4%, R^2^ = 0.9597)

log(DMPs/TMPs) = 0.71x − 0.55 (0.9% < EasyRo% < 3.4%, R^2^ = 0.9884)

log(MTR) = 0.84x − 0.75 (0.9% < EasyRo% < 3.4%, R^2^ = 0.9706)

These significant positive correlations are strong argument for using log(DMPs/TMPs), log(MPs/P) and log(MTR) as maturity parameters, especially for mature to over-mature source rocks.

The relationship between log(MPs/P) and EasyRo% could be divided into two stages, which are a slight slope of 0.19 within the EasyRo% range of 0.9% ∼ 2.1%, and a severe slope of 0.64 within the EasyRo% range of 2.1% ∼ 3.4% (Fig. 6a). This suggests that the log(MPs/P) is more sensitive at EasyRo% above 2.1%. Although, higher R^2^ and slopes for log(DMPs/TMPs) and log(MTR) than for log(MsP/P) were observed (Fig. 6), the abundance of DMPs and TMPs are commonly very low in over-mature samples, especially for TMPs (Fig. 2), so that it was very difficult to get the values of log(DMPs/TMPs) and log(MTR). On the contrary, relative high abundance of P and MPs in over-mature samples (Fig. 2) provides a possible mean for assessing over-mature source rocks.

Unfortunately, all four proposed equations have not been examined by natural geological maturity sequence. Concerning this fact and the difference between EasyRo% and natural maturity level, all above four equations and its application could be optimized by more pyrolysis experiments and investigation of natural samples.

## 4 Conclusions

A simulation pyrolysis experiment of bitumen in the vessels was carried out. The yields and distributions of Ps and evolution characteristics of some aromatic maturation parameters have been discussed. Several maturity parameters based on the distribution of Ps have been discussed. The main achievement is that two novel and an optimized maturation parameters based on the distribution of Ps are proposed: log(MPs/P) = 0.19x + 0.08 (x = EasyRo%) (0.9% < EasyRo% < 2.1%); log(MPs/P) = 0.64x − 0.86 (2.1% < EasyRo% < 3.4%); log(DMPs/TMPs) = 0.71x − 0.55 (0.9% < EasyRo% < 3.4%); log(MTR) = 0.84x − 0.75 (0.9% < EasyRo% < 3.4%). The significant positive correlations between proposed parameters and EasyRo% are strong argument for using log(MPs/P), log(DMPs/TMPs) and log(MTR) as maturity parameters, especially for mature to over-mature source rocks.

## Declarations

### Author contribution statement

Yongli Wang, Baoxiang Wu: Conceived and designed the experiments.

Gen Wang, Zepeng Sun, Liang Xu, Shenzheng Zhu: Performed the experiments.

Lina Sun, Zhifu Wei: Contributed reagents, materials, analysis tools or data.

Zixiang Wang: Performed the experiments; Analyzed and interpreted the data; Wrote the paper.

### Funding statement

This work was supported by the Chinese Academic of Sciences Key Project (Grant No. XDB10030404 and XDB03020405), the National Programs for Fundamental Research and Development of China (973 Program) (Grant No.2012CB214700), the National Science Foundation (41572350, 41172169, 41272147, 40672123), Western Light Joint Scholars Project and the Key Laboratory Project of Gansu Province (Grant No.1309RTSA041).

### Competing interest statement

The authors declare no conflict of interest.

### Additional information

No additional information is available for this paper.

## Figures and Tables

**Table 1:** The yields of aromatic hydrocarbons and relative abundances of Ps, P, MPs, DMPs and TMPs.

Temperatures (°C)	EasyRo (%)	Aro* (mg/g)	[Ps] (%)	[P] (%)	[MPs] (%)	[DMPs] (%)	[TMPs] (%)
335	0.7	20.77	41.45	31.71	20.67	25.77	21.86
359	0.9	54.07	37.95	12.42	19.89	34.71	32.98
383	1.1	54.43	39.07	12.49	25.39	38.65	23.48
407	1.4	7.99	40.98	14.97	34.89	36.94	13.20
432	1.7	11.10	40.68	14.80	40.80	35.48	8.92
456	2.1	9.68	38.92	18.51	52.40	25.24	3.85
480	2.5	3.10	24.81	10.39	61.41	26.57	1.63
504	3.0	3.18	10.65	4.00	60.01	35.00	0.99
527	3.4	3.67	4.08	2.37	44.60	52.50	0.53

* Aro: the yield of aromatic hydrocarbons.

**Table 2:** Identification of peaks from Fig. 2.

Peak number	Assignment	Abbreviation	Alkyl series
1	phenanthrene	P	P
2	3-methylphenanthrene	3-MP	MPs
3	2-methylphenanthrene	2-MP
4	9-methylphenanthrene	9-MP
5	1-methylphenanthrene	1-MP
6	3-ethylphenanthrene	3-EP	DMPs
7	2- + 9-ethylphenanthrene; 3,6-dimethylphenanthrene	2-EP;9-EP;3,6-DMP
8	3,5- + 2,6-dimethylphenanthrene	3,5-DMP;2,6-DMP
9	2,7-dimethylphenanthrene; 2-ethylphenanthrene	2,7-DMP;2-EP
10	2,10- + 3,9- + 3,10-dimethylphenanthrene	2,10-DMP;3,9-DMP;3,10-DMP
11	2,5-dimethylphenanthrene	2,5-DMP
12	1,7-dimethylphenanthrene	1,7-DMP
13	2,3-dimethylphenanthrene	2,3-DMP
14	1,9- + 4,9-dimethylphenanthrene	1,9-DMP;4,9-DMP
15	1,8-dimethylphenanthrene	1,8-DMP
16	1,2-dimethylphenanthrene	1,2-DMP
17	1,3,6- + 1,3,10- + 2,6,10-trimethylphenanthrene; 2-ethylphenanthrene;5-methylphenanthrene	1,3,6-TMP;1,3,10-TMP;2,6,10-TMP;2-EP;5-MP	TMPs
18	1,3,7- + 2,6,9- + 2,7,9-trimethylphenanthrene; 7-ethylphenanthrene;1-methylphenanthrene	1,3,7-TMP;2,6,9-TMP;2,7,9-TMP;7-EP;1-MP
19	1,3,9- + 2,3,6-trimethylphenanthrene	1,3,9-TMP;2,3,6-TMP
20	1,6,9- + 1,7,9- + 2,3,7-trimethylphenanthrene	1,6,9-TMP;1,7,9-TMP;2,3,7-TMP
21	1,3,8-trimethylphenanthrene	1,3,8-TMP
22	2,3,10-trimethylphenanthrene	2,3,10-TMP
23	C3-phenanthrene	C_3_-P
24	1,6,7-trimethylphenanthrene	1,6,7-TMP
25	1,2,6-trimethylphenanthrene	1,2,6-TMP
26	1,2,7- + 1,2,9-trimethylphenanthrene	1,2,7-TMP;1,2,9-TMP
27	1,2,8-trimethylphenanthrene	1,2,8-TMP

**Table 3:** Values of phenanthrene maturity parameters at each temperature point.

Temperatures (°C)	EasyRo%	MPI 1	MPI 2	MPI 3	[P_1_]	PAI 1	MDR	MTR	log(MPs/P)	log(DMPs/TMPs)	log(MTR)
335	0.7	0.52	0.62	1.88	13.14	0.65	0.80	0.95	−0.19	0.07	−0.02
359	0.9	0.85	1.18	1.43	4.71	1.60	0.57	0.60	0.20	0.02	−0.22
383	1.1	1.00	1.31	1.47	4.88	2.03	0.66	1.08	0.31	0.22	0.03
407	1.4	1.42	1.85	2.27	6.14	2.33	0.94	2.64	0.37	0.45	0.42
432	1.7	2.08	2.53	3.81	6.02	2.76	1.15	4.57	0.44	0.60	0.66
456	2.1	2.72	3.39	6.85	7.20	2.83	2.08	13.61	0.45	0.82	1.13
480	2.5	3.78	4.56	5.14	2.58	5.91	2.31	37.74	0.77	1.21	1.58
504	3.0	3.02	3.96	2.48	0.43	15.01	1.71	60.78	1.18	1.55	1.78
527	3.4	1.57	1.98	1.17	0.10	18.86	0.85	84.27	1.28	2.00	1.93
